# Compact Holographic Projection Display Using Liquid-Crystal-on-Silicon Spatial Light Modulator

**DOI:** 10.3390/ma9090768

**Published:** 2016-09-09

**Authors:** Wei-Feng Hsu, Ming-Hong Weng

**Affiliations:** Department of Electro-Optical Engineering, National Taipei University of Technology, Taipei 10608, Taiwan; baalism2@gmail.com

**Keywords:** LCoS spatial light modulator, holographic imaging, beam shaping, speckle reduction

## Abstract

This paper presents a holographic projection display in which a phase-only spatial light modulator (SLM) performs three functions: beam shaping, image display, and speckle reduction. The functions of beam shaping and image display are performed by dividing the SLM window into four sub-windows loaded with different diffractive phase elements (DPEs). The DPEs are calculated using a modified iterative Fourier transform algorithm (IFTA). The function of speckle reduction is performed using temporal integration of display images containing speckles. The speckle contrast ratio of the display image is 0.39 due to the integration of eight speckled images. The system can be extended to display full-color images also by using temporal addition of elementary color images. Because the system configuration needs only an SLM, a Fourier transform lens, and two mirrors, the system volume is very small, becoming a potential candidate for micro projectors.

## 1. Introduction

The framework of digital holography can be presented as a three-stage process: hologram acquisition, hologram processing, and hologram reconstruction [1]. Hologram reconstruction, the output stage of real and virtual optical information, always fascinates people because of the visual effects it provides, for example, in 3D motion pictures. In hologram reconstruction techniques, holographic projection displays (HPDs) have gained considerable attention as a two- and three-dimensional projection display option [2,3,4,5,6,7,8,9,10]. A key component to implementing the HPD is the spatial light modulator (SLM), which consists of a great number of electronically driven pixels [11]. The SLM serves to change the amplitude and phase of the incident light. The light is typically produced by semiconductor optical sources such as light emitting diodes and laser diodes. The use of lasers as an optical source in HPD provides a number of advantages, including good color performance, high contrast, high brightness, and coherence. However, the speckle issue associated with this approach has been difficult to overcome. Although the use of an incoherent optical source can effectively reduce the effects of speckle [9,10], it decreases the overall efficiency of light utilization or the image quality because the operations of optical waves in HPD are on the basis of complex-valued magnitude, a nature of coherent optics.

Many methods have been proposed to decrease the effects of speckle, including moving the diffuser [12], optimizing the diffuser [13], rotating the screen [14], vibrating a light pipe [15], and moving diffractive optical elements [16]. Unfortunately, these methods consume too much power, and mechanical rotation and high-frequency vibration make the system unstable, resulting in a decrease of coherence. One method was proposed to reduce the effects of speckle in a diffractive image through the integration of multiple speckled images using an SLM [2,17]. Some systems even require two SLMs for speckle reducing and projection imaging [7]. However, the cost of the extra devices or two SLMs required for such systems is too high. In this work, we configure a holographic display using a single SLM (PLUTO: HOLOEYE Photonics AG, Berlin-Adlershof, Germany), which performs three functions: beam shaping, image display, and speckle suppression.

When performing the beam shaping of laser sources in the proposed HPD, both the pixel dimension of the SLM and the diameter of the laser beam are small enough to generate far-field diffraction within the volume of the system without using any optical lenses. Similarly, in image projection display, diffraction of the wavefront modulated by the SLM changes the rectangular optical fields into specific patterns. Small SLM pixels are required because the beam dimension is several times larger than that of beam-shaping beams. It can be achieved without optical lenses needed, especially when SLM pixel is much smaller. This makes it possible to use only a few additional optical components to construct the entire system within a very limited volume. The size is suitable for handheld projectors or head mounted displays.

## 2. Holographic Display Using a Single SLM

### 2.1. System Schematic

**Figure 1:**
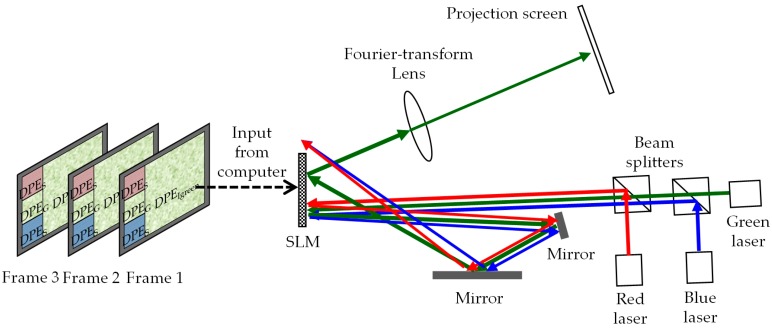
Schematic diagram of the diffractive projection display system. When a green image is produced, for example, the green laser beam becomes rectangular as it reflects back to the spatial light modulator (SLM). The diffractive phase element, DPE_Igreen_, in sub-window W_I_ modulates the rectangular beam and produces a display image in the projection screen. The red and blue beam-shaping DPEs diffract the laser beams away from the window W_I_. Using temporal integration of multiple speckled images reduces the speckle effect in the display image.

Figure 1 presents a schematic diagram showing the proposed display system. Optical sources include red (650 nm), green (533 nm), and blue (450 nm) solid state lasers for the emission of laser beams (diameters of approximately 2 mm) impinging directly on the SLM through beam splitters. The horizontal incident angle of the laser beam is 2.5°. A polarizer and an analyzer (not shown in Figure 1 for simplicity) ensure that the polarization of the optical beam is coincidental to the alignment of the liquid-crystal molecules in the SLM. The modulated beams are reflected by the SLM, and then reflected two more times by mirrors before returning to the SLM at an angle of approximately 15°. The shape of the second incident wavefront is rectangular, completely covering the right part of the SLM window, which is loaded with diffractive phase element (DPE) of the display image. The diameters of the first incident beam are sufficiently small to eliminate the need for optical lenses to perform far-field diffraction of the reflected beams. The beam, reflected by two mirrors, turns into a rectangular shape. Then, it is modulated again by the SLM. The display image is generated through a Fourier-transform lens in the form of a Fraunhofer pattern of the modulated wavefront. Note that only an optical lens is required in the current configuration of the system, and no lenses will be required in the near future when the SLM pixel pitch becomes small enough that far-field diffraction can be obtained over a short distance.

### 2.2. Beam Shaping of Laser Sources

**Figure 2:**
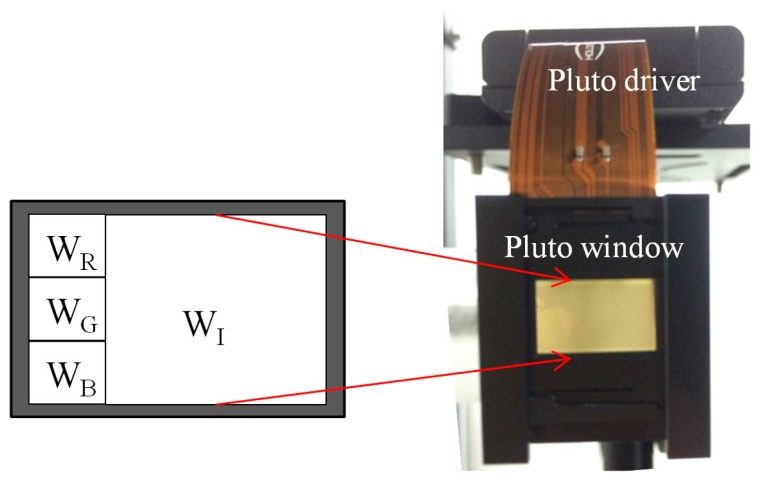
Sub-window partition for beam shaping and imaging DPE display.

The first function performed by the SLM is the shaping of beams from incident lasers. The elliptical cross section of the incident beam is changed to rectangular by the SLM [7]. The left side of the SLM window is divided into three regions, each of which contains 360 × 360 pixels. The region is referred to as the sub-window of beam shaping. The area of the sub-window is approximately 2.9 × 2.9 mm^2^, which is just enough to accommodate the profile of the laser beam. The red/green/blue (R/G/B) lasers are incidental to three sub-windows W_R_, W_G_, and W_B_, sequentially from top to bottom. Each sub-window modulates a laser beam to generate light with a rectangular intensity distribution covering the right side of the SLM window W_I_, called the image sub-window, as shown in Figure 2. The display of colors is conducted sequentially as red, green, and blue. This means that each SLM frame generates a color image while the other two laser beams are diffracted away from the SLM window. For example, when a green image is meant for projection, the green sub-window is loaded with a diffractive phase element (DPE_G_) for beam shaping and the red and blue sub-windows are loaded with a binary phase element DPE_S_. These result in the generation of green light with a rectangular intensity distribution in the image sub-window and the two other beams are directed away using DPE_S_. When a blue image is required, a blue rectangle is generated in the image sub-window and the red and the green beams are directed away. The same operation occurs for the red laser beam.

**Figure 3:**
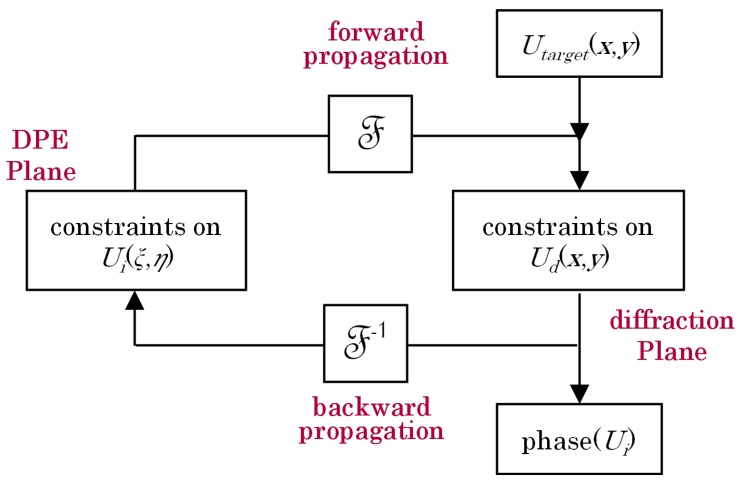
Schematic diagram of iterative Fourier transform algorithm (IFTA).

The DPEs were designed using modification of the iterative Fourier transform algorithm (IFTA) [18,19]. The IFTA consists of an operation loop with a Fourier transform and an inverse Fourier transform for the forward and the reversal operations, respectively. Two nodes connect the two operations: one node is for the field constraint in the DPE plane and the other node is for the diffraction plane, as shown in Figure 3. The IFTA begins with an initial optical field distribution *U_d_*(*x*, *y*) composed of the target amplitude *U_target_*(*x*, *y*) and a random phase. The complex-valued function *U_d_*(*x*, *y*) represents the field distribution in the diffraction plane. In our system, the amplitude of *U_d_*(*x*, *y*) can be a rectangular pattern for beam shaping DPEs and a display image for image projection in the later section. An inverse Fourier transform of *U_d_*(*x*, *y*) is performed to generate the optical field distribution *U_i_*(*ξ*, *η*) in the DPE plane, given by [20]

(1) $$U_{i}(\xi ,\eta )\propto \int_{-\infty }^{\infty }\int_{-\infty }^{\infty }U_{d}(x,y)\cdot e^{j\frac{2\pi }{\lambda z}(x\xi +y\eta )}dxdy$$

where *λ* is the operating wavelength. Parameter *z* is the round-trip distance from the beam-shaping to imaging sub-windows. In the projection of holographic images, *z* becomes the foal length of the Fourier-transform lens. The amplitude of *U_i_*(*ξ*, *η*) is changed to a constant profile to accommodate the optical incident field. The field function *U_d_*(*x*, *y*) in the diffraction plane is then calculated by performing a Fourier transform of the modified *U_i_*(*ξ*, *η*), given by

(2) $$U_{d}(x,y)\propto \int_{-\infty }^{\infty }\int_{-\infty }^{\infty }U_{i}(\xi ,\eta )\cdot e^{-j\frac{2\pi }{\lambda z}(x\xi +y\eta )}d\xi d\eta$$

Calculations of the diffraction efficiency and uniformity of the intensity distribution of *U_d_*(*x*, *y*) are used to evaluate the performance of *U_i_*(*ξ*, *η*). The amplitude of *U_d_*(*x*, *y*) is replaced by the target *U_target_*(*x*, *y*) and the phase distribution remains unchanged. This operation forces the intensity distribution of diffractive image to approach the target image. The operations of Fourier transform and inverse Fourier transform completing one loop of IFTA are iteratively conducted until *U_d_* reaches stagnation. The phase distribution of the final *U_i_*(*ξ*, *η*) is a local solution of DPE to produce a diffractive image resembling the pattern of the target amplitude *U_target_*(*x*, *y*).

Producing a full-color image through the temporal addition of elementary RGB color images was achieved by modifying the IFTA to produce DPEs of different wavelengths, due to the fact that the SLM gamma cannot be changed in time the way that elementary color images can be. We select a gamma curve that would result in 2π phase retardation of green light at 533 nm, scaled using 256 levels. Thus, the green DPEs were designed using a normal IFTA program. Blue images were created by calculating DPEs using a normal IFTA program with its target image scaled up to the ratio of blue to green wavelengths, approximately 0.844. Phase retardation is nearly 2.37π, scaled down using the inverse of 0.844; therefore, the phase value of blue DPEs is quantized into 216 gray levels, which are obtained as follows: 256 × *λ*_blue_/*λ*_green_. Fewer quantization levels and thus larger phase steps of the blue DPEs do not significantly affect the diffraction efficiency and the noise of the diffractive images because the total number of 216 phase levels is large enough to make the quantization errors insignificant. Red images are produced by modifying the IFTA using the stepwise quantization method [21] to compress the phase retardation to a range from 0 to 1.7π, which is then scaled down 2π according to the wavelength ratio of green light to red light. The phase value is then quantized into 256 levels. The narrow phase range of the red DPEs causes an on-axis spot in the diffraction image and reduces the diffraction efficiency by approximately 20%. The lowered intensity of the diffractive pattern results in a low signal and relatively high noise levels, i.e., a small signal-to-noise ratio of the image.

**Figure 4:**
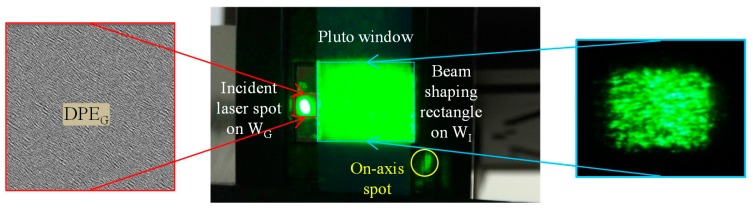
Beam-shaping rectangle (green) with a size the same as that of image sub-window W_I_.

In the shaping of the laser beam, the target images of three colors contain an off-axis rectangle, the size of which is identical to the image sub-window W_I_. When an elliptical laser beam is incident on the rectangular DPE_G_, a low-pass filtering effect occurs on the diffractive pattern, rather than changing the shape of the diffractive pattern. It might be the reason that the speckled diffractive rectangle can still produce the target images. The off-axis rectangle is arranged to avoid on-axis reflection of the other two colors, even though most of their power has been diffracted to ±1st orders using a binary phase grating in the corresponding sub-windows. Figure 4 represents a photo of SLM window in which the diffractive rectangle from a 533 nm laser is shaped by DPE_G_ of 360 × 360 pixels. The gray levels from 0 to 255 in DPE_G_ represent the phase modulation of from 2π to 0 onto the green incident wavefront. Despite considerable speckling in the diffractive rectangle, it still results in an optical incidence for DEP_I_, loaded in W_I_, which is a diffractive phase element to generate the desired projection image.

### 2.3. Display of Holographic Images

**Figure 5:**
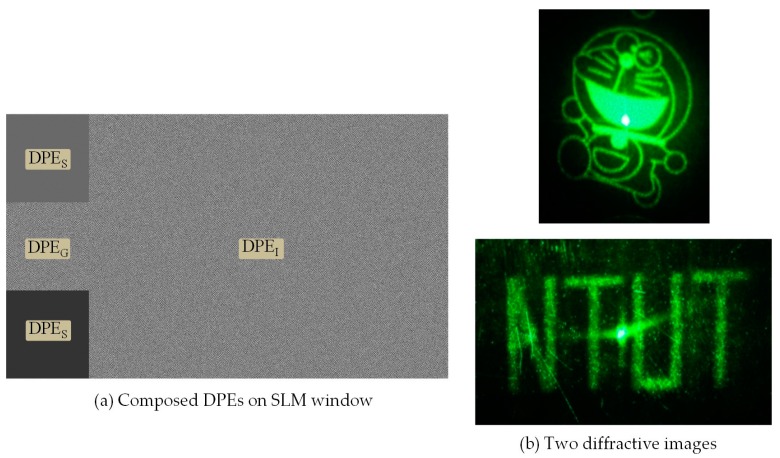
(**a**) Composed DPE and (**b**) two display images diffracted from DPE_I_.

The second function performed by the SLM is diffractive imaging using an optical Fourier transform system. Loaded in sub-window W_I_, the phase element DPE_I_ (1560 × 1080 pixels), which is used to generate the display images, is calculated using the IFTA program. This is the same program used for the beam shaping DPE. The projection of two images is shown in Figure 5. Despite the fact that the optical wave incident to W_I_ is non-uniform and contains speckles, the quality of the diffractive image of the illuminated DPE_I_ is close to that illuminated by uniformly plane waves. Speckles always exist [22], as outlined in the following section.

The imaging in Figure 5, which was obtained from the diffraction of DPE_I_, differs from what was expected. The imaging distance exceeds the focal length of the Fourier transform lens and the image size is larger than the theoretical result. We speculate that this is due to the fact that the rectangular waves illuminating DPE_I_ were divergent, rather than planar. The beam-shaping DPE (360 × 360 pixels) is smaller than the imaging DPE (1560 × 1080 pixels). Such divergent illumination is equivalent to having a point source at the extension from W_I_ to W_R/G/B_. Thus, the Fraunhofer image was obtained at the image point of the pseudo point source [20] (p. 120), resulting in the magnification of the image.

### 2.4. Speckle Reduction

The third function performed by SLM involves speckle reduction through the temporal integration of numerous statistically independent specked images. The speckle phenomenon in an image can be evaluated by speckle contrast *C*, defined as [23]

(3) $$C=\frac{\sigma_{I}}{\bar{I}}$$

where *σ_I_* and *Ī* are the standard deviation and the mean of the speckle intensity of image, respectively. Based on the definition given above, the speckle contrast of an image is the reciprocal of the signal-to-noise ratio (SNR), which is an important measure to evaluate the quality of the image ([23], p. 28). One can simply reverse the value of *C* to create the SNR of the image. When a total of *N* images are added up, the speckle contrast ratio *r* of the resultant image is given by [24]

(4) $$r=\frac{C_{R}}{C_{avg}}$$

where *C_R_* and *C_avg_* are the speckle contrast of the resultant image and the arithmetic average of the *N* original speckle contrast values, respectively. When *N* speckled images are statistically independent, the speckle seen in the resultant image is reduced and *r* is equal to 1/$\sqrt{N}$.

When using the IFTA program to design DPEs, a random phase array and the amplitude of the target image compose the initial complex-valued field function. When using different random phase arrays to compose the initial field function of the IFTA, the resulting DPEs generate diffractive images possessing similar amplitudes and random phases. Using different initial phases results in a set of speckled images with intensities that are statistically independent [17]. The sum of these images results in an image with a lower speckle contrast and higher image quality. We believe that the use of different DPE design methods and quantized phase levels do not change the randomness of phase distribution of the diffractive image, and, therefore, would not considerably affect the speckle contrast. The effective method to reduce the speckles in HPD is the temporal integration of multiple speckled images.

**Figure 6:**
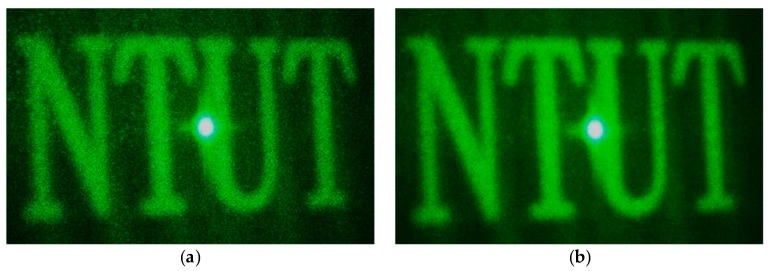

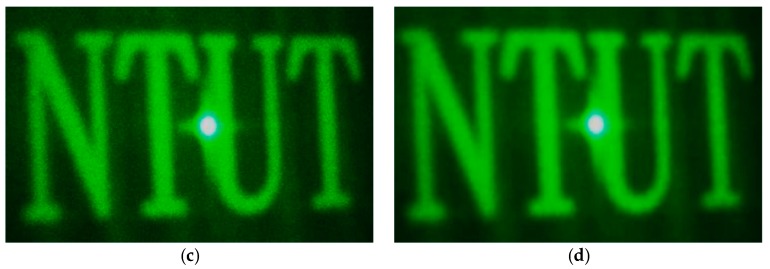
Resultant images of the integration of (**a**) one; (**b**) two; (**c**) four; and (**d**) eight diffractive images.

This resulted in 16 DPEs for the 533 nm laser source, the diffractive images of which were captured using a digital camera, as shown in Figure 6a. We then randomly selected two, four, and eight DPEs, which were queued up in a cyclic order for display by the SLM. The exposure time of the camera was set to capture at least one cycle of diffractive images. As shown in Figure 6, the appearance of speckles in the diffractive image was gradually reduced as more speckled images were combined.

**Figure 7:**
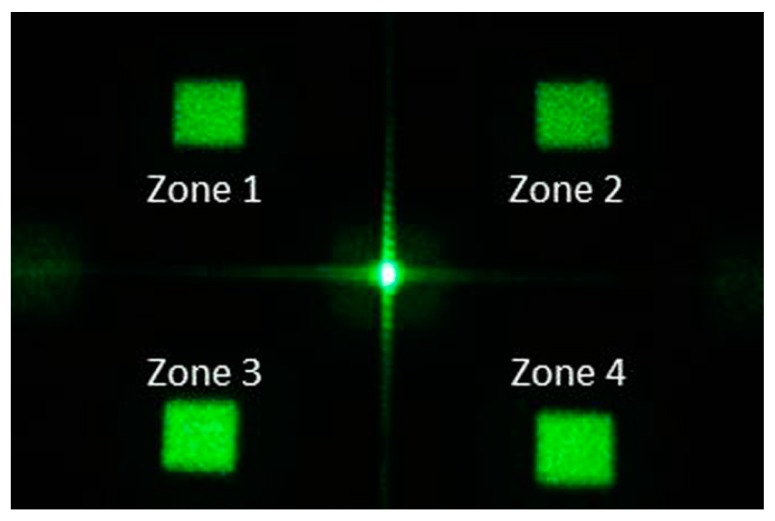
Diffractive image used to evaluate the speckle contrast of integrated images.

To evaluate the efficacy of this speckle reduction method, we had another target image in which four rectangles are located off-axis, as shown in Figure 7. As in the previous experiment, we obtained 16 DPEs and the diffractive images and then calculated the correlation coefficients of these images, selecting those close to zero. As in the previous experiment, the selected DPEs were queued and displayed by the SLM and the resultant images were captured.

**Figure 8:**
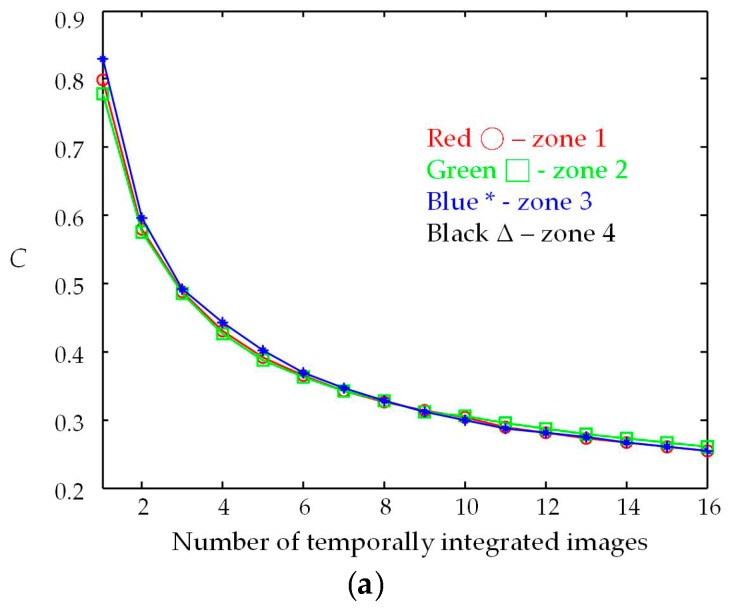

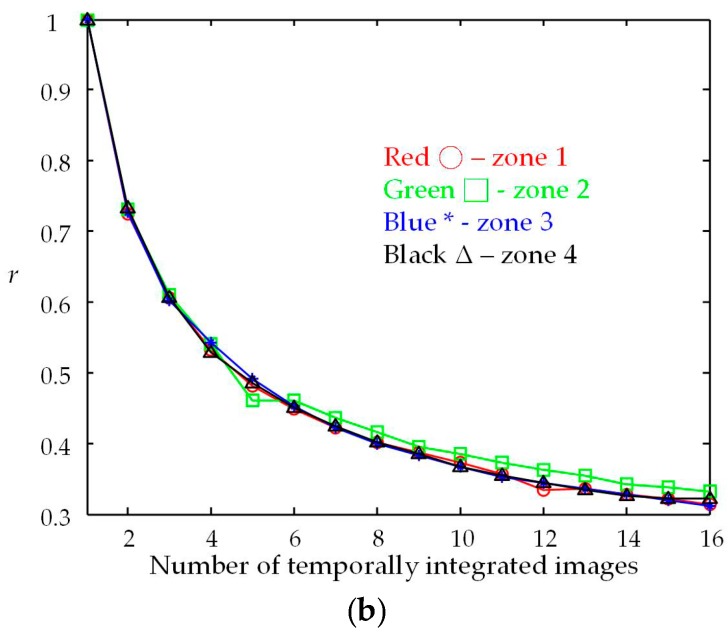
(**a**) Speckle contrast and (**b**) speckle contrast ratio of resultant images using temporal integration of speckled images.

The test target shown in Figure 7 contains four squares, ordered Zone 1 to 4, located in four quadrants of the image space. The calculated speckle contrasts *C* of the squares in the resultant images is shown in Figure 8a. The reduction in *C* was very close among the four squares, despite differences in the number of integrated images, thereby illustrating that this effect was uniform throughout the image space. As shown in Figure 8b, the speckle contrast ratios (r) were slightly higher than the theoretical result, 1/$\sqrt{N}$. This may be due to the fact that the selected images are not statistically independent. In addition, crosstalk occurred when the DPEs were displayed consecutively on SLM, resulting in correlation between display images. The speckle contrast ratios of the resultant images were 0.39 for *N* = 8 (*C* = 0.32) and 0.31 for *N* = 16 (*C* = 0.26). The results are of the same magnitude as the speckle contrasts of despeckled images using a moving diffuser [25].

## 3. Conclusions

In this article, we present a holographic projection display scheme that requires very limited volume because it needs only a phase-only SLM, a Fourier-transform lens, and two mirrors. The beam shaping and image display functions were performed by dividing the SLM window into four sub-windows. Speckle reduction and color combination were achieved by displaying the DPEs in temporal sequence. The DPEs were calculated using a modification of IFTA. The phase range of DPE and grayscale of the DPE patterns were adjusted for different wavelengths to fix the gamma of the SLM and simplify system operations. Beginning with a random-phase field function, the IFTA program produces DPEs with diffractive images that are nearly statistically independent. Speckle in the resultant image was reduced through the temporal integration of the diffractive images. Eight and sixteen images were used to produce speckle contrast values of 0.32 and 0.26, respectively. The speckle contrast ratios in these images were 0.39 and 0.31, respectively.

The presented system is a prototype of holographic projection display. The future work includes:

1. Producing full-color imaging by temporal integration of elementary red/green/blue images.
2. Increasing the efficiency of speckle reduction and decrease the number of the integrated speckled images by integrating the negatively correlated speckle images [18].
3. Producing virtual images from the diffractive phase elements.
4. Using fibers to introduce laser light.

## References

[1] Tsang P.W.M., Poon T.-C. (2016). Review on the state-of-the-art technologies for acquisition and display of digital holograms. IEEE Trans. Ind. Inform..

[2] Cable A.J., Buckley E., Mash P., Lawrence N.A., Wikinson T.D., Crossland W.A. (2004). Real-time binary hologram generation for high-quality video projection applications. Proc. SID Int. Symp. Dig. Tech. Pap..

[3] Jesacher A., Maurer C., Schwaighhofer A., Bernet S., Ritsch-Marte M. (2008). Near-perfect hologram reconstruction with a spatial light modulator. Opt. Express.

[4] Palima D., Glückstad J. (2008). Comparison of generalized phase contrast and computer generated holography for laser image projection. Opt. Express.

[5] Buckley E. (2011). Holographic laser projection. J. Disp. Technol..

[6] Zheng H.D., Yu Y.J., Wang T., Asundi A. (2012). Computer generated kinoforms of real-existing full-color 3D objects using pure-phase look-up-table method. Opt. Lasers Eng..

[7] Chang Y.-S., Hsu W.-F., Hsu K.-H., Lin H.Y. (2014). Full-frame projection displays using a liquid-crystal-on-silicon spatial light modulator for beam shaping and speckle suppression. Appl. Opt..

[8] Zhao Y., Cao L., Zhang H., Tan W., Wu S., Wang Z., Yang Q., Jin G. (2016). Time-division multiplexing holographic display using angular-spectrum layer-oriented method. Chin. Opt. Lett..

[9] Kim Y.S., Kim T., Woo S.S., Kang H., Poon T.-C., Zhou C. (2013). Speckle-free digital holographic recording of a diffusely reflecting object. Opt. Express.

[10] Liu J.-P., Guo C.-H., Hsiao W.-J., Poon T.-C., Tsang P. (2015). Coherent experiments in single-pixel digital holography. Opt. Lett..

[11] Sun J., Wu S.-T. (2014). Recent advances in polymer network liquid crystal spatial light modulators. J. Polym. Sci. B Polym. Phys..

[12] Kubota S., Goodman J.W. (2010). Very efficient speckle contrast reduction realized by moving diffuser device. Appl. Opt..

[13] Trisnadi J.I. (2004). Hadamard speckle contrast reduction. Opt. Lett..

[14] Shin S.C., Yoo S.S., Lee S.Y., Park C.-Y., Park S.Y., Kwon J.W., Lee S.-G. (2006). Removal of hot spot speckle on rear projection screen using the rotating screen system. J. Disp. Technol..

[15] Sun M., Lu Z. (2010). Speckle suppression with a rotating light pipe. Opt. Eng..

[16] Wang L., Tschudi T., Halldórsson T., Pétursson P.R. (1998). Speckle reduction in laser projection systems by diffractive optical elements. Appl. Opt..

[17] Hsu W.-F., Yeh C.F. (2011). Speckle suppression in holographic projection displays using temporal integration of speckle images from diffractive optical elements. Appl. Opt..

[18] Wyrowski F., Bryngdahl O. (1988). Iterative Fourier-transform algorithm applied to computer holography. J. Opt. Soc. Am. A.

[19] Poon T.-C., Liu J.-P. (2014). Introduction to Modern Digital Holography: With Matlab.

[20] Goodman J.W. (2005). Introduction to Fourier Optics.

[21] Hsu W.-F. (2005). Backward iterative quantization methods for designs of multilevel diffractive optical elements. Opt. Express.

[22] Wyrowski F., Bryngdahl O. (1989). Speckle-free reconstruction in digital holography. J. Opt. Soc. Am. A.

[23] Goodman J.W. (2007). Speckle Phenomena in Optics: Theory and Applications.

[24] Hsu W.-F., Chu I.-L. (2011). Speckle suppression by integrated sum of fully developed negatively correlated patterns in coherent imaging. Prog. Electromagn. Res. B.

[25] Kuratomi Y., Sekiya K., Satoh H., Tomiyama T., Kawakami T., Katagiri B., Suzuki Y., Uchida T. (2010). Speckle reduction mechanism in laser rear projection displays using a small moving diffuser. J. Opt. Soc. Am. A.

